# Inhibition of Tumor Lymphangiogenesis is an Important Part that EGFR-TKIs Play in the Treatment of NSCLC

**DOI:** 10.7150/jca.35448

**Published:** 2020-01-01

**Authors:** Yan Zhang, Xinying Yang, Hongchun Liu, Minghui Cai, Yang Shentu

**Affiliations:** 1 Division of Antitumor Pharmacology, State Key Laboratory of Drug Research, Shanghai Institute of Materia Medica, Chinese Academy of Sciences, Shanghai, 201203, China.; 2 Department of Thoracic Surgery, Shanghai Chest Hospital, Shanghai Jiao Tong University, Shanghai, 200030, China.; 3 Department of Thoracic Surgery, Taizhou hospital of Zhejiang province, Zhejiang, 317000, China.

**Keywords:** EGFR-TKIs, NSCLC, lymphangiogenesis, JAK/STAT3, LYVE-1

## Abstract

Epidermal growth factor receptor (EGFR) tyrosine kinase inhibitors (TKIs) have been widely used to treat non-small cell lung cancer (NSCLC) because they inhibit tumour growth and metastasis. However, the underlying mechanisms are not fully understood. Here, we investigate whether anti-lymphangiogenesis mechanisms contribute to the anti-tumour effects of EGFR-TKIs. Three different EGFR-TKIs (Gefitinib, Afatinib, and AZD9291) were used to determine the possible biological effects of EGFR-TKIs on lymphangiogenesis *in vitro* and *in vivo*. EGFR-TKIs inhibited human lymphatic endothelial cells (HLEC) proliferation, migration and tube formation at the indicated concentrations. Conditioned medium from human lung adenocarcinoma HCC827 cells treated with EGFR-TKIs also inhibited HLEC migration and tube formation. EGFR-TKIs inhibited VEGFC secretion, which further influenced HLEC behaviour *in vitro*. Afatinib inhibited tumour growth and lymphangiogenesis in the HCC827 xenograft mouse model. The densities and tube diameters of the lymphatic vessels were decreased in a dose-dependent manner, as shown by lymphatic vessel endothelial hyaluronan receptor 1 (LYVE-1) staining. EGFR-TKIs also inhibited the expression of important lymphangiogenesis regulatory factors vascular endothelial growth factor 2/3 (VEGF2/3), VEGFC, and chemokine receptor 7 (CCR7) as shown by immunocytochemistry (IHC) staining. Additional assays confirmed that the JAK/STAT3 signalling pathways play important roles in the anti-lymphangiogenesis process induced by EGFR-TKIs. Inhibition of lymphangiogenesis is another important role that the three EGFR-TKIs play in the treatment of lung cancer and the Janus kinase/signal transducers and activators of transcription 3 (JAK/STAT3) maybe an important signalling pathway regulating lymphangiogenesis, which provides a new idea for clinical therapy of lung cancer.

## Introduction

Lung cancer is the most common cancer and the leading cause of cancer death in both men and women 1. The incidence of NSCLC accounts for 80% to 85% of lung cancer, and NSCLC is a primary disease type with increasing incidence and mortality rates 2. Recurrence and metastasis are tremendous obstacles to NSCLC therapy and result in poor prognosis and high fatality. Cancer cells may alter the normal pattern of lymphangiogenesis, thus creating fast tracks for tumour dissemination and distant metastasis. Therefore, therapeutic strategies for NSCLC that focus on lymphangio-genesis inhibition are very important.

EGFR is highly expressed on the surface of a considerable proportion of NSCLC cells 3. Lymphangiogenesis plays a vital role in tumour dissemination, almost equal to the action of angiogenesis 4. With the successful application of three generations of TKIs (Gefitinib, Afatinib, AZD9291) to patients with NSCLC, we wondered whether the lymphangiogenesis process may also be affected by EGFR-TKIs treatment. Therefore, we performed experiments to find possible new mechanisms of action of EGFR-TKIs to better guide the clinical application of EGFR-TKIs in cancer therapy.

Several cytokines and their receptors, such as VEGFC, VEGFD, and VEFGR2/3, are involved in the process of lymphatic vessel growth 5. CCL21/CCR7 plays an essential role in lymphangiogenesis in pancreatic cancer and breast cancer, and this action promotes VEGFC secretion and lymphatic metastasis and reduces survival rates 6, 7. CCR7 is overexpressed in NSCLC. Whether CCR7 suppresses lymphangiogenesis to mediate EGFR-TKI's therapeutic effects on NSCLC patients remains unknown. LYVE-1 is a predictor of lymphatic vessel formation in tumours. The JAK-STAT3-VEGFC pathway is involved in lymphangiogenesis and tumour growth and invasion in gastric cancer 8. Here, the major lymph-angiogenetic factor VEGFC, the CCL21/CCR7 chemokine axis and the JAK/STAT3/VEGFC pathway were studied during EGFR-TKI treatment *in vitro* and *in vivo*.

Tumour metastasis is the main cause of death in cancer patients 9. Entering the lymphatic vessels is the first step of cancer cell lymphatic dissemination from the primary tumour to the distal organs. Inhibiting lymphangiogenesis and subsequently blocking the metastasis route are highly important for suppressing tumour development. Understanding the underlying mechanism of tumourigenesis and lymphangiogenesis will provide a valuable strategy for cancer therapy. EGFR-TKIs are widely used in the therapy for lung cancer patients, especially NSCLC patients. Despite the anti-angiogenesis effects of EGFR-TKIs, no studies on the effects of the EGFR-TKIs Gefitinib, Afatinib, and AZD9291 on lymphangiogenesis in lung cancer have been reported. Therefore, we determined the possible mechanism of these three effective EGFR-TKIs in lymphangiogenesis and tumour metastasis in NSCLC models *in vitro* and *in vivo*.

## Materials and methods

### Reagents

3-(4, 5-dimethylthiazol-2-yl)-2, 5-diphenyltetrazolium bromide (MTT) (5 mg/mL; Sigma, St. Louis, MO, USA), dimethyl sulfoxide (DMSO; Sigma). Afatinib (BIBW2992) was purchased from Selleck Chemicals (Houston, TX). All these compounds were dissolved in DMSO at 10 mmol/L as stock solutions and stored at -20°C. Antibodies against GAPDH were from Epitomics (Burlingame, CA). Antibodies against CCR7 (Y59), VEGFR3, VEGFR2, VEGFC, and LYVE-1 were from Abcam (Cambridge, MA, USA). Antibodies against phospho-EGFR (Y1068), EGFR, Akt, phospho-Akt (S473), JAK1/2, phospho-JAK1 (Tyr1022/1023), phospho-JAK2 (Tyr1007/1008), c-Myc, STAT3, phospho-STAT3 (Tyr705), FAK, and phospho-FAK (Tyr925) were purchased from Cell Signaling Technology (Beverly, MA).

### Cell lines

HLECs and human NSCLC cell line HCC827: HLECs were purchased from CellBiologics (Chicago, IL, USA) and maintained in complete human endothelial cell medium or endothelial cell base medium (CellBiologics; Chicago, USA) containing 10% fetal bovine serum (FBS) (Gibco; USA), 100 ng/mL endothelial cell growth supplement (CellBiologics; Chicago, IL, USA) and penicillin/streptomycin (BasalMedia, Shanghai, China). HCC827 cells were kindly gifted by Dr. Pasi A. Jӓnne (Dana-Farber Cancer Institute, Boston, MA) and cultured in RPMI 1640 (Corning, USA) containing 10% FBS and glucose. HCC827 cells were authenticated by short tandem repeat (STR) fingerprinting. All the cells were incubated at 37°C in a humidified atmosphere of 5% CO_2_ and 95% air.

### Cell proliferation assay

HLEC proliferation was assessed using MTT assays. Cells were seeded in 96-well plates at a density of 10000 cells per well and allowed to attach overnight. The indicated concentrations of EGFR-TKIs were added to the cells for 24 hours and 48 hours. Each concentration was replicated in three wells. Then, the cells were treated with 20 µl MTT (5 mg/mL) for 4 hours at 37°C, and 100 µl premixed solution (10% sodium dodecyl sulfate (SDS), 5% isobutanol, and 0.01 M HCL) was added. The absorbance was measured at 570 nm by a spectra-MAX190 (Molecular Devices, Sunnyvale, CA).

### Cell migration assays

We applied transwell inserts (Corning, NY) to study cell migration activity. HLECs (10^5^ cells/well) suspended in 100 µl serum-free medium were added to the upper chambers, and conditioned medium from HCC827 cells treated with or without EGFR-TKIs was added to the lower chambers. The medium in the upper and lower chambers was removed after 18 hours of treatment. The cells were fixed in 90% ethanol for 30 minutes at 4°C. The unfixed cells were removed using cotton swabs, and the fixed cells were stained with a 0.1% crystal violet solution for 30 minutes. Finally, images were obtained with a fluorescence microscope (Olympus, Japan). The crystal violet stain was further dissolved in 10% acetic acid and measured at 600 nm using a spectra-MAX190 to obtain quantitative results.

### Tube formation assays

Ninety-six-well plates and pipettes were precooled at 4°C overnight. The plates were coated with 55 µl liquid Matrigel (Corning, NY) per well. The entire process was performed on ice, and bubbles were avoided. The plates were incubated for 10 minutes at 40°C, and the Matrigel was polymerized for 30 minutes at 37°C. HLECs (2×10^4^ cells/well) suspended in 100 µl endothelial cell medium (ECM) were added to 96-well plates. Then, the cells were treated with varying concentrations of EGFR-TKIs (6.25 μM, 12.5 μM and 25 μM) for 4 hours, and images were obtained by fluorescence microscopy.

### Western blotting assays

HCC827 cells (3.5×10^5^ cells/well) or HLECs (3.0×10^5^ cells/well) were added to 6-well plates. After the cells reached 80% confluence, the medium was replaced, and the cells were treated with EGFR-TKIs for the indicated hours. The cells and tumour tissues were lysed with SDS, and the protein concentrations were determined with a bicinchoninic acid protein assay kit. The samples were denatured at 100°C for 15 minutes and run on SDS-PAGE gels. The membranes were probed with primary and horseradish peroxidase-conjugated secondary antibodies, and bands were detected using enhanced chemiluminescence (ECL Plus; Pierce).

### Enzyme-linked immunosorbent assay

HLECs (3×10^5^ cells/well) were added to 6-well plates and grown to 80% confluence. The medium was then replaced with fresh medium. The cells were treated with or without EGFR-TKIs (6.25 μM, 12.5 μM and 25 μM) for 4 hours. The resultant cell supernatants were used for ELISA. HCC827 cells (3.5×10^5^ cells/well) were added to a 6-well plate overnight. Conditioned medium treated with the indicated concentrations of EGFR-TKIs for 9 hours or 24 hours was added to the cells. VEGFC levels in the supernatants were determined with a human VEGFC ELISA kit (Westang, Shanghai, China).

### Immunohistochemical analysis

Sections were dewaxed and antigens were retrieved in dimethylbenzene and a graded ethanol series. The slides were incubated with phosphate-buffered saline (PBS) three times for five minutes and then quenched with fresh 3% H_2_O_2_ for 10 minutes and washed with PBS. Nonspecific binding was blocked by incubating the tissue sections with 10% normal goat serum in PBS for 15 minutes. After rinsing with PBS, the tissue slides were incubated with rabbit anti-human LYVE-1 (1:200), CCR7 (1:600), VEGFR3 (1:50), VEGFR2 (1:50), VEGFC (1:50), or VEGF (1:200) at 4°C overnight. Then, the slides were rinsed with PBS, incubated at 37°C for 20 minutes with horseradish peroxidase goat anti-rabbit polyclonal antibodies and rinsed again with PBS. The sections were stained with diaminobenzidine (DAB) solution. Proteins with brown staining were considered positive.

### Animal studies

HCC827 cells (5 × 10^6^) were injected subcutaneously into the right flanks of nude mice. After passaging once, tumour tissues were inoculated into the mice. After the tumours grew to approximately 200 mm^3^, the mice were divided into different groups according to their tumour volumes and body weights. Afatinib (1.25, 2.5, 5, and 10 mg/kg) was administered to the mice for 28 days. Tumour volume (TV) was calculated with the following formula: V = (a × b^2^)/2 (a, width; b, length). The individual relative tumour volume (RTV) was calculated as follows: RTV = V_t_/V_0_, where V_t_ is the volume on each day, and V_0_ represents the volume at the beginning of the treatment. Blood and tumour tissues were collected at the end of the experiment.

### Statistical analysis

All data were analysed by GraphPad Prism v.6 (La Jolla, CA).

## Results

### EGFR-TKIs inhibit HLEC proliferation, migration and lymphatic tube formation

**Figure 1:**
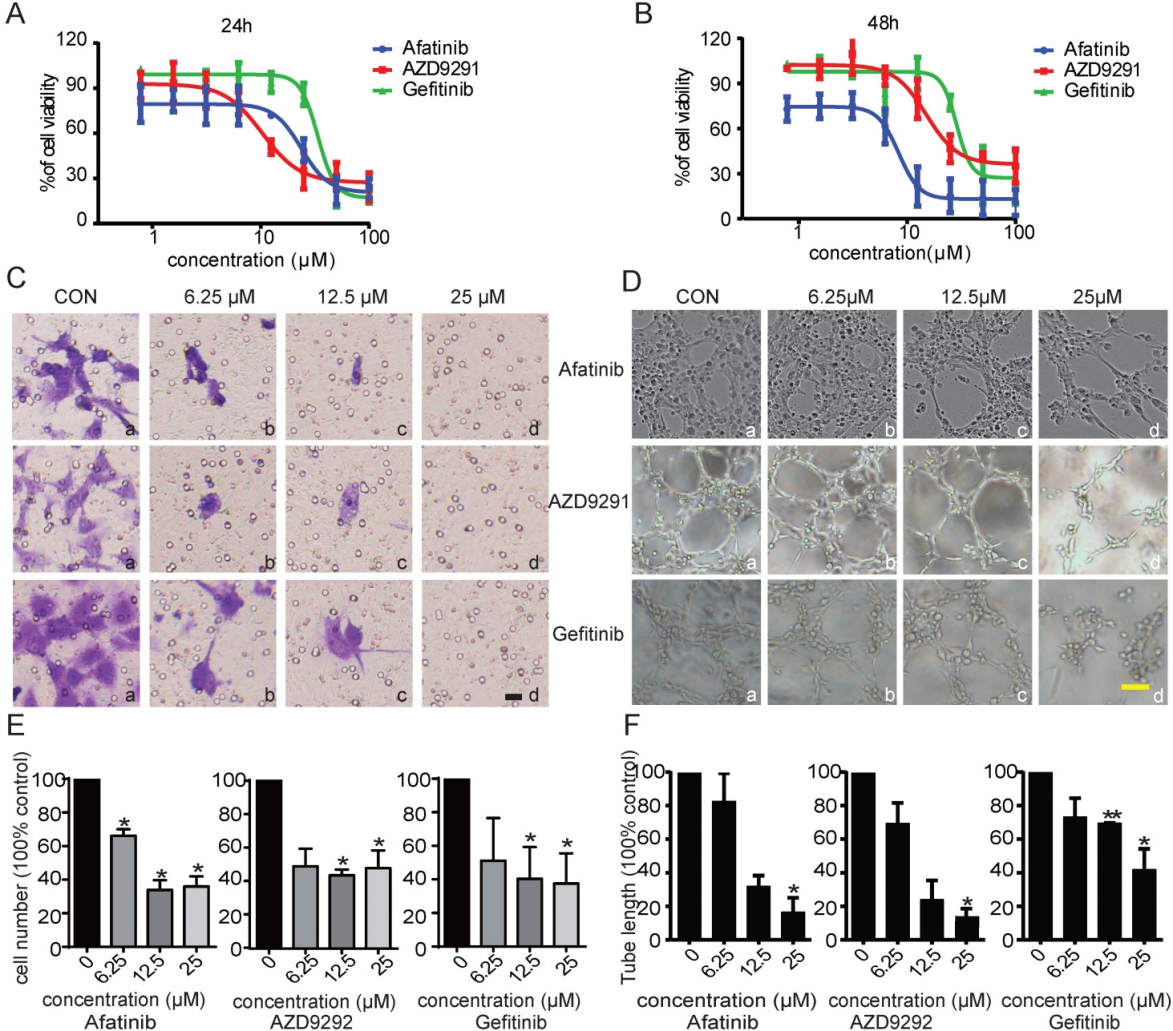
** Inhibitory effects of the three EGFR-TKIs on HLEC proliferation, migration and tube formation.** A. HLECs were exposed to increasing concentrations (1 μM - 100 μM) of EGFR-TKIs for 24 hours and 48 hours. Cell proliferation was assessed by MTT assay. The IC_50_ values for Afatinib, AZD9291, and Gefitinib in HLECs after 24 hours were 25.20 ± 1.09 μM, 15.7 ±4.6 μM, and 36.1 ± 5.3 μM, respectively. B. HLEC proliferation was detected at 48 hours under the same experimental conditions as in A. The IC50 values for the three EGFR-TKIs were 11.7 ± 3.12 μM, 11.8 ± 4.3 μM, and 29.1 ± 1.54 μM. C. EGFR-TKIs inhibited HLEC migration. HLECs were seeded in Boyden chambers and treated with different concentrations of Afatinib, AZD9291 and Gefitinib (6.25, 12.5 and 25 μM). Serum was used to induce HLEC migration from the upper chamber to the lower chamber. Images (20x magnification) were obtained at 24 hours. The scale bar equals 100 μM. D. EGFR-TKIs inhibited HLEC tube formation. HLECs were plated on Matrigel in the absence or presence of different concentrations of Afatinib, AZD9291 and Gefitinib. Images were taken at 4 hours after plating. E and F, Statistical analysis of the migration and tube formation assay data shown in panels C and D. The scale bar equals 100 μM. *p < 0.05, the results are the mean ± SD.

Angiogenesis and lymphangiogenesis are two important aspects of tumour development and metastasis. It has been reported that EGFR inhibitors exhibit prominent anti-angiogenesis and anti-cancer activities. However, no research has focused on whether the anti-tumour metastasis activity of EGFR-TKIs occurs through inhibiting lymphangiogenesis in NSCLC. In this study, we first investigated the possible effects of EGFR-TKIs on tumour lymphangiogenesis. HLECs were treated with EGFR-TKIs at various concentrations for 24 hours and 48 hours. We found that three generations of EGFR-TKIs potently inhibited HLEC proliferation in a dose-dependent manner. The IC_50_ values of the three inhibitors at 48 hours were 29.1 ± 1.54 µM (Gefitinib), 11.7 ± 3.12 µM (Afatinib), and 11.8 ± 4.3 µM (AZD9291), respectively (Figure 1, A and B). Cell migration contributes to cancer metastasis. Thus, transwell assays were used to examine the inhibitory effect of the three EGFR-TKIs on HLEC migration. As shown in Figure 1C, EGFR-TKIs strongly inhibited HLEC migration, even at a concentration of 6.25 µM, suggesting an inhibitory effect of EGFR-TKIs on HLEC motility.

To further examine the anti-lymphangiogenic activity of EGFR-TKIs, we applied tube formation assays to determine the influence of EGFR-TKIs on lymphangiogenesis in HLECs. We found that tubes began to form after HLECs were incubated on the surface of Matrigel for 4 hours. Compared to control treatment, EGFR-TKIs suppressed tube formation indistinctively at a dose of 6.25 µM. However, at concentrations of 12.5 µM and 25 µM, the three inhibitors reduced tube formation with limited numbers of enclosed cells, which indicated their direct inhibitory effect on lymphangiogenesis (Figure 1D). Statistical analyses showed the significance of the inhibitory effects of EGFR-TKIs to HLECs' motility and lymph vessels formation (Figure 1E and F). All of the above results confirmed the anti-lymphangiogenic and anti-metastatic effects of EGFR-TKIs *in vitro*.

### Conditioned medium from EGFR-TKI-treated HCC827 cells inhibits HLEC migration and lymphatic tube formation

**Figure 2:**
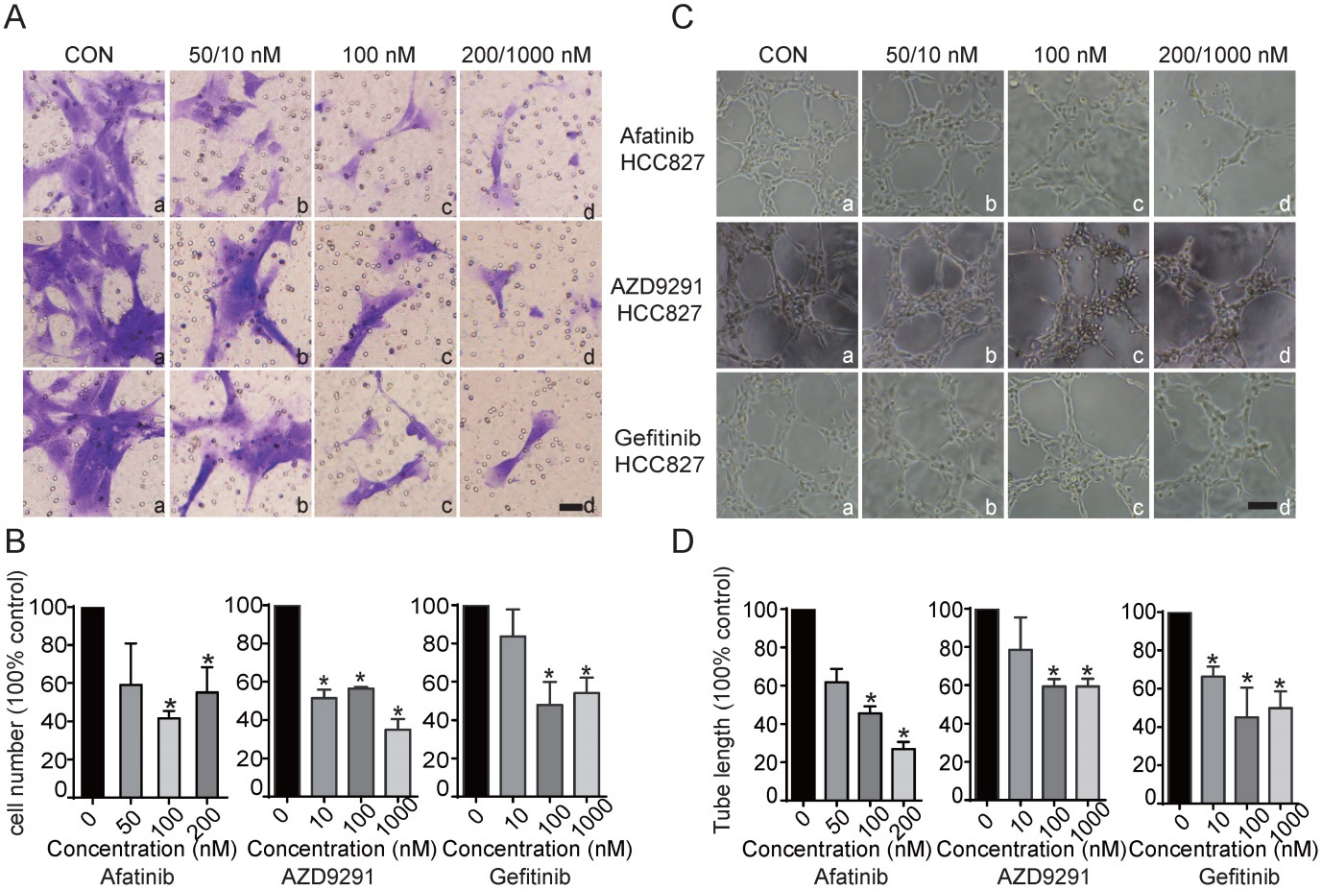
** Inhibitory effects of conditioned medium from HCC827 cells treated with EGFR-TKIs on HLEC migration and tube formation.** A. Conditioned medium from HCC827 cells treated with EGFR-TKIs for 18 hours suppressed HLEC migration. The scale bar equals 200 μM. B. Cells that migrated to the lower chamber were counted. Significance was found as indicated in the figures. *p < 0.05, the results are the mean ± SD. C. EGFR-TKI-treated HCC827 cell conditioned medium inhibited HLEC tube formation. The scale bar equals 100 μM. D. Tube length was calculated, and significance is shown in the figure. Conditioned medium from HCC827 cells inhibited HLEC tube formation. *p < 0.05, the results are shown as the mean ± SD.

For this experiment, supernatants from HCC827 cells treated with/without EGFR-TKIs were harvested and used as conditioned medium to determine the possible influence on HLEC migration and tube formation. HCC827 cells were first treated with EGFR-TKIs for 24 hours. Then, the supernatants were collected and added to the bottom chamber. VEGFC (10 ng/mL) was added to the bottom chamber at the same time. HLECs were added to the upper chamber and incubated for 18 hours, and the migrated cells were stained. We found that supernatants from EGFR-TKI-treated HCC827 cells remarkably inhibited HLEC migration (Figure 2A and B). To determine whether the tumour environment can influence lymphatic tube formation, culture medium consisting of 40% conditioned medium from HCC827 cells and 60% endothelial cell culture medium (ECM) supernatant was used to suspend HLECs. Then, the cells were plated on 96-well plates coated with Matrigel. We found that the tube length of the lymphatic vessels was markedly reduced by supernatants from cells treated with EGFR-TKIs, even at a concentration of 10 nM (Figure 2C and D). The results indicated that cytokines secreted into the medium may affect HLEC motility and lymphatic vessel formation.

### EGFR-TKIs inhibited VEGFC secretion in HCC827 cells

**Figure 3:**
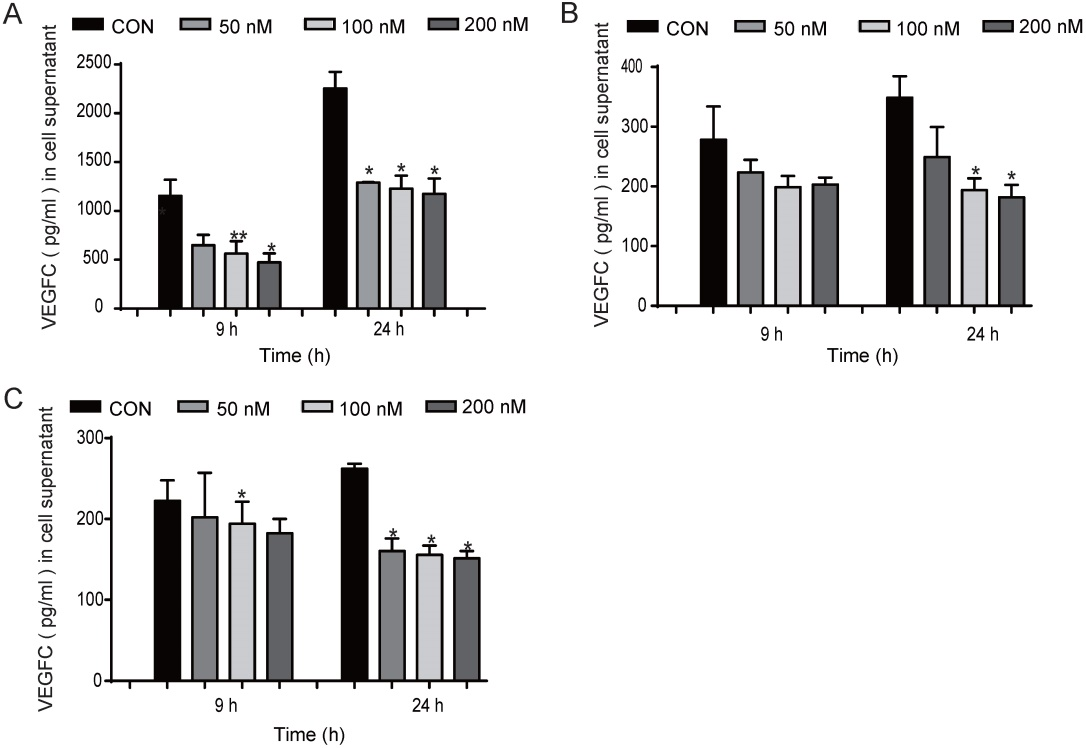
** Inhibited VEGFC secretion in HCC827 cells at different concentrations and time points.** EGFR-TKIs inhibited VEGFC secretion in HCC827 cells as shown in A. (Afatinib), B.(AZD9291), and C. (Gefitinib) after treatment for 9 and 24 hours. *p < 0.05, **p < 0.01, the results are shown as the mean ± SD.

We found that EGFR-TKIs and the conditioned medium of HCC827 cells treated with EGFR-TKIs could affect HLEC lymphangiogenesis. Therefore, we hypothesize that EGFR-TKIs themselves and some cytokines secreted into the supernatant may participate in the inhibition process. VEGFC is one of the most important lymphangiogenesis inducers. Its overexpression in cancer cells modulates lymphangiogenesis and stimulates metastasis 10. Therefore, in this experiment, we detected the expression of VEGFC under different conditions. We found that treatment with the three inhibitors Afatinib, Gefitinib, and AZD9291 at 100 nM reduced VEGFC secretion from HCC827 cells at 24 hours. The inhibitory effects also appeared after the cells were treated for 9 hours (Figure 3, A, B and C). Taken together, these data indicated that EGFR-TKIs mediate lung cancer cell VEGFC expression, which further regulates lymphatic vessel formation.

### EGFR-TKIs inhibited the JAK/STAT3 and FAK signalling pathways and c-Myc, VEGFR2, and VEGFR3 expression in HCC827 cells

**Figure 4:**
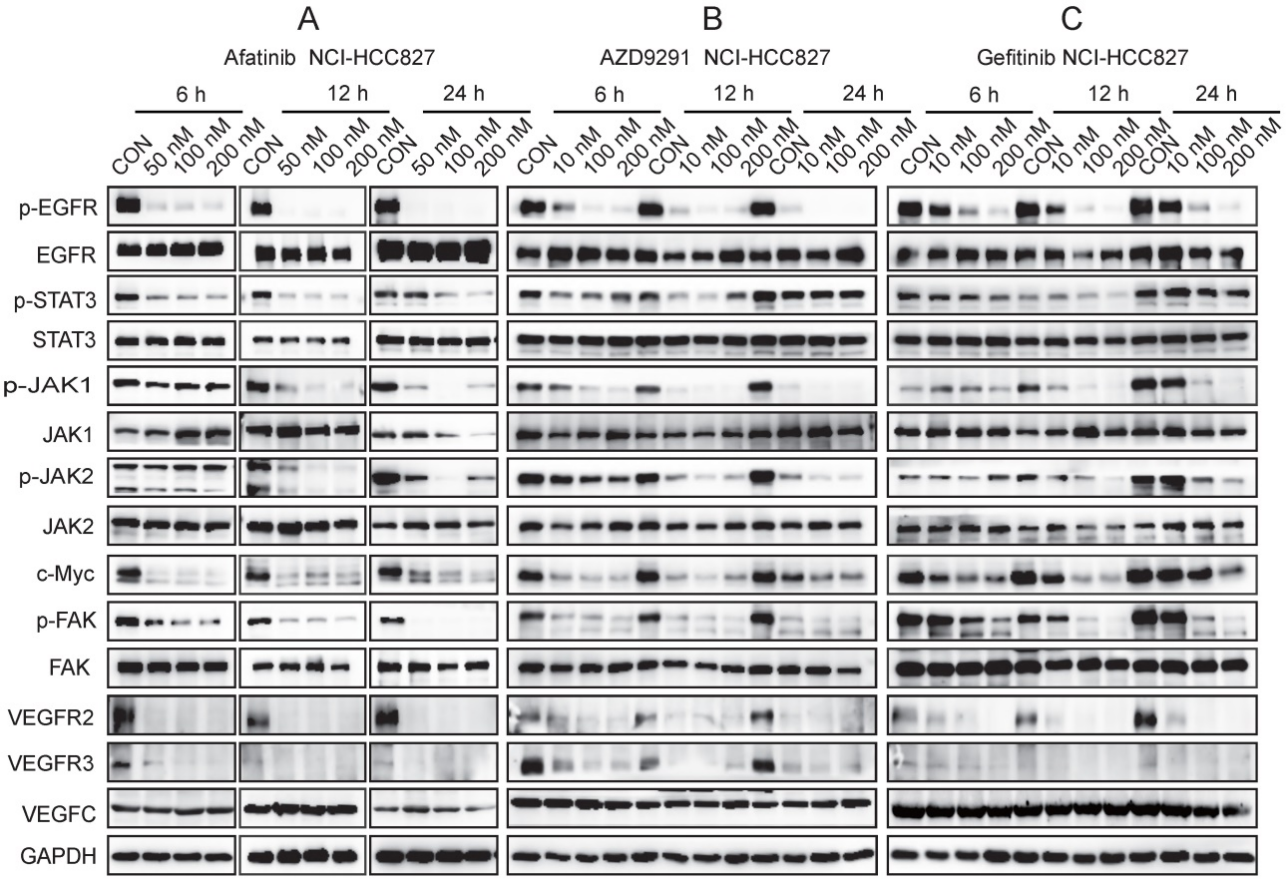
** JAK/STAT3, FAK, and c-Myc are involved in the signalling pathway mediated by EGFR-TKIs in HCC827 cells.** HCC827 cells were treated with different EGFR-TKIs at different doses and for different times. Western blot analyses were performed after the cells were harvested at 6 hours, 12 hours, and 24 hours. A. HCC827 cells were cultured overnight. Afatinib was added to the cells at 50 nM, 100 nM, and 200 nM. B. The effects of AZD9291 on HCC827 cell signalling pathways were determined by Western blotting. C. Gefitinib inhibited the JAK/STAT3, FAK, and c-Myc signalling pathways in HCC827 cells. GAPDH was used as an endogenous control.

JAK1, 2/STAT3 are highly expressed in NSCLC, and their activation correlates with increased VEGF and bFGF expression^11^. VEGFC and its receptors VEGFR2 and VEGFR3 play important roles in cancer development and metastasis. VEGFC/VEGFR2 is an important target for anti-angiogenesis therapy in cancer. VEGFC has a high affinity for VEGFR3, which is expressed in lymphatic endothelial cells, and stimulates the associated intracellular signalling cascades to promote lymphangiogenesis. In our experiment, we found that EGFR-TKIs inhibited VEGFR2 and VEGFR3 expression in HCC827 cells; therefore, we further analysed the JAK1,2/STAT3 pathway. The three inhibitors Gefitinib, Afatinib, and AZD9291 inhibited the activation of JAK1, JAK2, STAT3 and FAK in HCC827 cells, as well as the phosphorylation of EGFR, and these effects are characteristic of EGFR inhibitors (Figure 4). Overexpression of the c-Myc gene is closely related to uncontrolled cell growth, division and metastasis 12. The three EGFR-TKIs inhibited the expression of c-Myc, which reduced NSCLC cell proliferation and metastasis to lymph vessels (Figure 4). In conclusion, the JAK1,2/STAT3 signalling pathway, FAK phosphorylation, and c-Myc were involved in the mechanism of EGFR-TKIs in HCC827 cells. This mechanism inhibited NSCLC cell movement to lymphatic vessels to prevent metastasis.

### EGFR-TKIs inhibited the JAK1,2/STAT3 signalling pathway and c-Myc expression in HLECs

**Figure 5:**
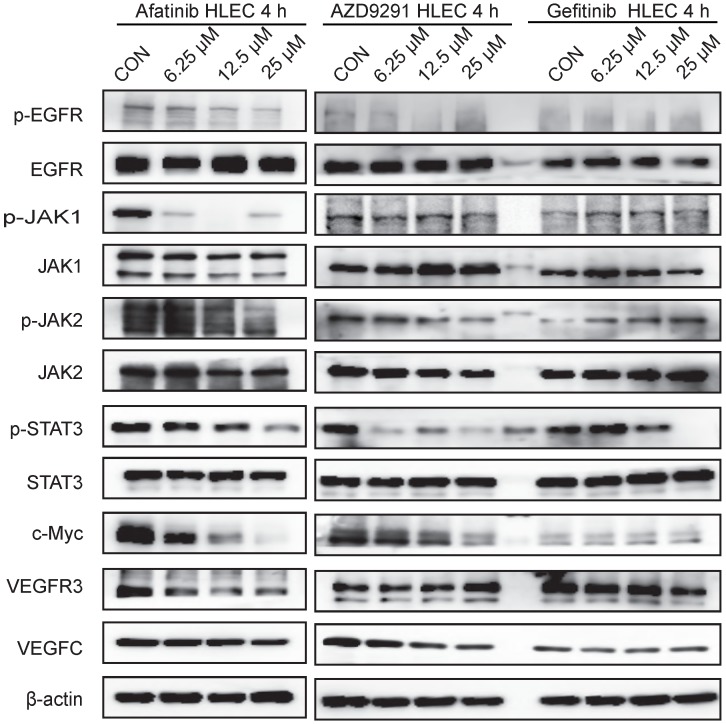
** EGFR-TKIs inhibit the JAK/STAT3 and c-Myc signalling pathways in HLECs.** HLECs were treated with three EGFR-TKIs at different concentrations (6.25, 12.5 25μM). Four hours later, the cells were collected and subjected to Western blot analysis. Afatinib, AZD9291 and Gefitinib inhibited the JAK1,2/STAT3 signalling pathway and reduced c-Myc expression. β-actin was used as an endogenous control.

HLECs were treated with EGFR-TKIs for 4 hours at different concentrations. All three inhibitors (Afatinib, AZD9291 and Gefitinib) inhibited the phosphorylation of EGFR, JAK1/2, STAT3, and c-Myc (Figure 5). Afatinib had some effects on VEGFR3 expression, and AZD9291 showed some inhibitory effects on VEGFC expression in HLECs. Through inhibiting the above signalling pathway, EGFR-TKIs can directly influence the proliferation and division of lymphatic cells and simultaneously reduce the ability of tumour cells to transfer to lymphatic vessels.

### *In vivo* experiments

#### Afatinib suppressed tumour growth in a dose-dependent manner

**Figure 6:**
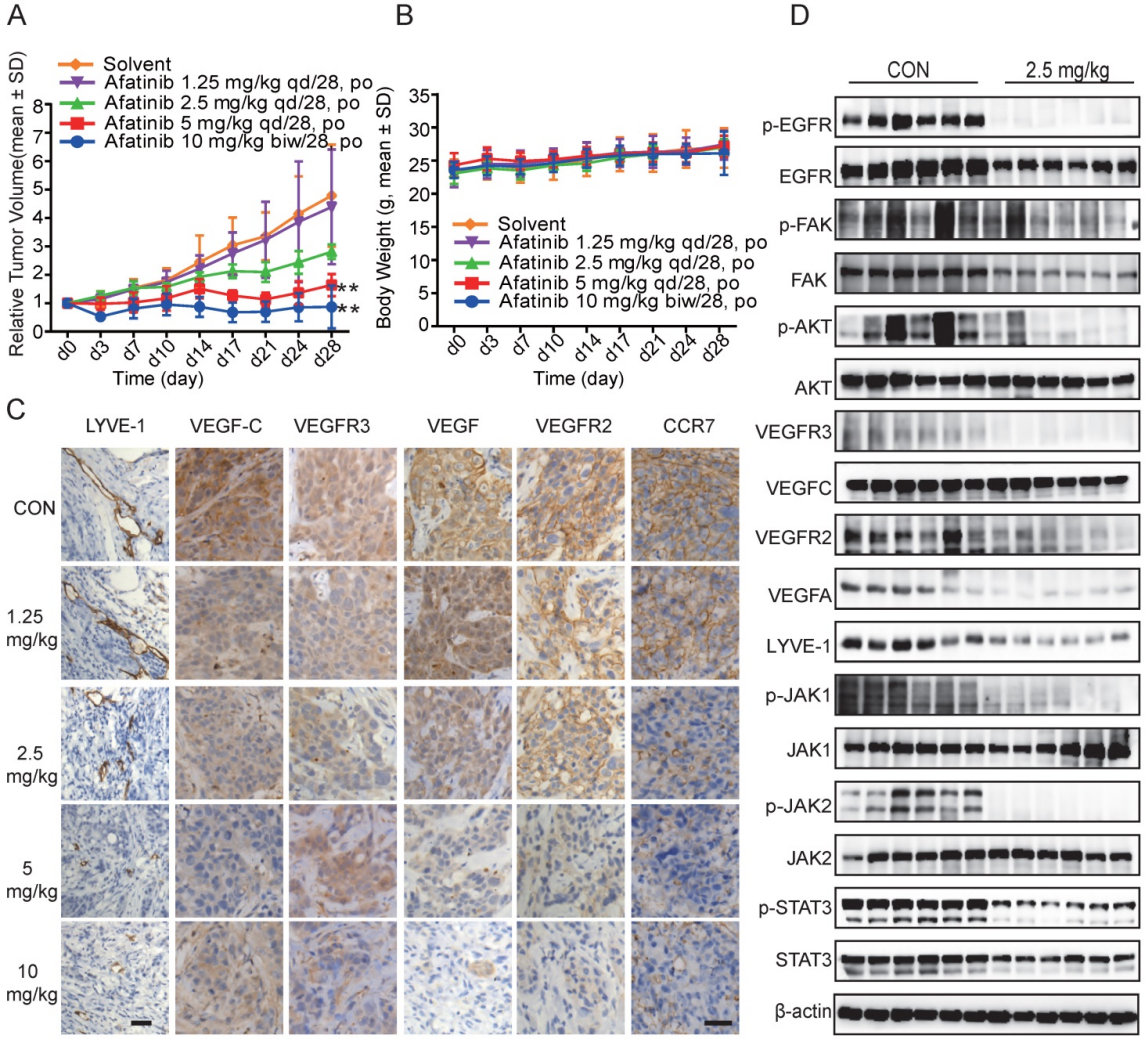
** EGFR-TKIs inhibited tumour growth, lymph tube formation and associated marker molecules in HCC827 tumour-bearing mice. HCC827 cells were harvested after cell growth and fused together.** Aliquots of 2 x 10^4^ cells were injected subcutaneously to create tumour-bearing mice. Mice bearing tumours were divided into seven groups after the tumour volumes reached 200-220 m^3^. There were 12 mice in the control group and 6 mice in each treatment group. The mice were administered Afatinib intraperitoneally at 1.25 mg/kg, 2.5 mg/kg, and 5 mg/kg once a day and then 10 mg/kg twice a week for 28 days. A. Compared to the control treatment, Afatinib significantly inhibited tumour growth at 5 mg/kg and 10 mg/kg. B. The body weights of each group were maintained at a steady state with a slight increase. C. Afatinib inhibited LYVE-1, VEGFC, VEGFR3, VEGF, VEGFR2, and CCR7 expression as detected by IHC staining. The scale bar equals 50 μM. D. Signalling pathways involved in the process of lymphangiogenesis with Afatinib treatment in HCC827 xenograft models. β-actin was used as an endogenous control.

Based on the results that Afatinib inhibited HLEC lymphangiogenesis, migration, proliferation, and tube formation directly and indirectly through conditioned medium from HCC827 cells *in vitro*, we further confirmed our observation *in vivo* using HCC827 tumour-bearing mice. Mice were divided randomly into different groups and treated with Afatinib at different doses (1.25 mg/kg, 2.5 mg/kg, and 5 mg/kg) daily for 28 days and with 10 mg/kg twice a week after grouping. Afatinib significantly inhibited tumour growth, with T/C 18.14% and 34.18% at doses of 10 mg/kg and 5 mg/kg, respectively. There was some inhibitory effect with T/C 58.74% at 2.5 mg/kg but no significant inhibitory effect at 1.25 mg/kg (Figure 6A). During the experiment, all mice were in good health, and no mice died. There was a slight increase in the body weights of the mice (Figure 6B).

#### Afatinib inhibited LYVE-1, VEGFR2, VEGFR3, VEGFC, VEGF, VEGFA and CCR7 expression and the JAK1,2/STAT3 and FAK signalling pathways in HCC827 xenograft tumour tissues

To explore whether the repression of lymphangiogenesis was associated with VEGFC, CCR7 and other relative proteins, we performed immunohistochemistry (IHC) staining using the antibodies. Afatinib inhibited LYVE-1, VEGFR2, VEGFR3, VEGFC, VEGF, and CCR7 expression in a dose-dependent manner in HCC827 xenograft tumour tissues, and these results demonstrated the anti-lymphangiogenic action of Afatinib (Figure 6C). We used LYVE-1 as a specific lymphangiogenesis marker to evaluate the effects of Afatinib on lymphatic vessel formation and found that Afatinib significantly reduced the number of lymphatic vessels. These results confirmed the lymphangiogenesis-inhibiting effect of Afatinib.

Further results from Western blotting assays using tumour tissues confirmed the mechanism of Afatinib on lymphangiogenesis that we found *in vitro*. Using total protein from HCC827 xenograft tumour tissues, we found that Afatinib inhibited the phosphorylation of JAK1,2, STAT3, and FAK, the protein expression of LYVE-1, VEGFR2, VEGFR3, VEGFC, VEGFA, and CCR7 (Figure 6D). These results agreed with the *in vitro* and IHC staining results. In conclusion, Afatinib inhibited the JAK1,2/STAT3 signalling pathway *in vivo* and suppressed the expression of VEGFC, which further suppressed the VEGFR2/3 signalling pathway. These findings indicate that EGFR-TKIs have anti-lymphangiogenic and anti-metastatic effects on NSCLC patients.

## Discussion

We found that three generations of EGFR-TKIs inhibited HLEC proliferation, migration and tube formation *in vitro*. Conditioned medium from HCC827 cells treated with EGFR-TKIs also reduced HLEC migration and lymphatic tube formation. EGFR-TKIs suppressed VEGFC secretion by HCC827 cells, which further inhibited HLEC behaviour. In tumour tissues, we also found reduced lymphatic vessel formation by detecting the expression of the lymphatic marker LYVE-1. Further *in vivo* and *in vitro* mechanistic studies showed that the JAK/STAT3 pathway contributed to the inhibitory effects of Afatinib. Our findings uncover new EGFR-TKI mechanisms of action for NSCLC therapy in which metastasis is inhibited through lymphangiogenesis inhibition effects.

In our experiments, we found that EGFR-TKIs inhibited HLEC proliferation, migration and tube formation, which validated the direct anti-lymphatic vessel formation effect of EGFR-TKIs *in vitro*. Conditioned medium from EGFR-TKI-treated HCC827 cells reduced lymphatic endothelial cell migration and lymphatic vessel tube formation. These results indicate that EGFR-TKIs might influence HCC827 cell cytokine secretion, which further affected HLEC migration and tube formation. VEGFC is the main growth factor through which lymphatic endothelial cells form the lymphatic network. According to the results we acquired, we hypothesized that the inhibited lymphatic network formation in NSCLC might be attributed to reduced VEGFC expression *in vitro* and *in vivo*. In this study, we subsequently detected VEGFC expression in the culture medium supernatants of HCC827 cells treated with EGFR-TKIs. After 24 h, the three inhibitors significantly inhibited the secretion of VEGFC by HCC827 cells; after 9 hours, we also observed an inhibitory effect, which in turn hindered the formation of lymphatic vessels in the tumours and slowed the lymphatic metastasis of lung cancer cells. VEGFC can disrupt the endothelial lymphatic barrier to promote colorectal cancer invasion 13. According to the results shown in Fig. 3, reduced VEGFC expression protected the lymphatic barrier and then reduced the number of lung cancer cells invading the lymphatic vessels and travelling to the distant lymph nodes. Lymph node metastasis is the source of distant metastases of deadly cancer 14, 15. In our study, we found the critical role that EGFR-TKIs play in suppressing the link between lymphatic metastasis and distant metastases in NSCLC models.

The JAK/STAT3 signalling pathway modulates cancer cell survival, proliferation, migration, invasion, angiogenesis, lymphangiogenesis and immune evasion in many types of cancers, such as prostate cancer, gastric cancer, and hepatocellular carcinoma 16, 17, 18. As shown in Figure 4, we found that EGFR-TKIs inhibited the phosphorylation of JAK1,2/STAT3, which mediated HCC827 cell proliferation, migration and invasion into lymphatic vessels. FAK promotes tumour development and metastasis through its effects on cancer cells and cells in the tumour microenvironment 19. The interaction of FAK and VEGFR-3 links FAK to lymphangiogenesis 20. In our experiment, the reduced phosphorylation of FAK in HCC827 cells indicated suppressed lung cancer cell growth and metastasis to the lymphatic vessels. Myc plays an important role in tumour initiation and maintenance 21. The decreased expression of c-Myc in HCC827 cells confirmed the anti-tumour effects of the three inhibitors, and in HLECs, this decrease indicated a reduced ability to build lymphatic vessels, as shown in Figure 4.5.

LYVE-1 was used as a lymphatic specificity biomarker. VEGFR-3 was the first lymphatic endothelial marker found by Alitalo 22. VEGFR-3 activation is one of the most important signals that regulates tumour-induced lymphangiogenesis 23, and VEGFR-3 is activated by VEGFC and VEGF-D, both of which are members of the VEGF family of growth factors 24. Using the NSCLC tumour model, we found that Afatinib suppressed the expression of LYVE-1, VEGFR2, 3, VEGFC, and VEGF in tumour tissues, indicating reduced lymphatic formation and possible VEGFC/VEGFR3 pathway involvement in the process. IHC staining analyses showed that LYVE-1 expression was reduced significantly by Afatinib treatment, which was consistent with the Western blot results. CCL21/CCR7 combined with VEGFC promotes lymphangiogenesis in pancreatic, breast, and lung cancer 6, 25, 26. In our experiment, EGFR-TKIs inhibited CCR7 protein levels, in addition to reducing VEGFC expression, and these results indicate the strong anti-lymphangiogenesis activity of EGFR-TKIs in NSCLC. Inhibited JAK1,2/STAT3 signalling pathway activation was also confirmed in EGFR-TKI-treated NSCLC tumour tissues. The* in vivo* results closely agreed with the data we acquired *in vitro*.

Taken together, these results demonstrated that EGFR-TKIs inhibited lung cancer progression and metastasis by suppressing lymphangiogenesis* in vitro* and* in vivo*. Understanding the interaction between lymphangiogenesis and tumour metastases may provide new clinical therapy strategies for cancer patients.
